# Justification of radiographic examinations: What are the key issues?

**DOI:** 10.1002/jmrs.211

**Published:** 2017-02-11

**Authors:** Jason Vom, Imelda Williams

**Affiliations:** ^1^ Faculty of Medicine, Nursing and Health Sciences, Department of Medical Imaging and Radiation Sciences (DMIRS) Monash University Clayton Australia

**Keywords:** Australian radiographers, barriers, decision making, justification

## Abstract

Justification of radiographic examinations is the practice of evaluating requested radiological examinations to assess for clinical merit and appropriateness based on clinical notes and patient information. This implies that justification in radiography requires the evaluation of requested examinations, the justification of exposures being applied and determining whether patients fit the recommended criteria for the procedure. Medico‐legal requirements by the professional registration body, the Medical Radiation Practice Board of Australia (MRPBA), identify justification as an advocated and obligatory practice for radiographers. Yet, justification remains an inconsistent practice implemented amongst Australian radiographers. This review aims to identify associated barriers inhibiting the consistent practice of justification and the hesitance by radiographers in practicing justification responsibilities. It also recommends a change in workplace culture which encourages radiographers to accept a more autonomous role that cultivates critical thinking, reflection and research‐informed decision making as justification will ultimately benefit patients.

## Introduction

The principle of justification is defined by the advisory body the International Commission on Radiological Protection (ICRP) as ‘any decision that alters the radiation exposure situation should do more good than harm’.^1^ ^(p.14)^ Before the radiographer applies ionising radiation to the patient, the acceptable and ethical practice of radiography should involve reviewing whether the benefits outweigh the risks associated with requested examinations.^1^ Therefore justification in radiography forms part of the duty of patient care in clinical practice and requires the evaluation and clarification of requested examinations.^2^ ^(p.33)^ Universally, medical radiation is a controllable source and should be applied on an individual basis to determine whether each patient fits the appropriate criteria for the diagnostic procedure.^3^ As a core task of the practitioner, justification needs to be applied to individual medical exposures consistently.^4^

The practice of justification within Australia's diagnostic setting is an important safeguard for patients against adverse health effects due to the application of ionising radiation.^1^, ^2^, ^4^ Internationally, and in particular in countries with regulatory bodies, recommendations are that all diagnostic requests should be justified.^5^ Justification is a necessary practice in radiography because the potential biological effects from x‐ray exposures can result in deleterious health effects as the risk of cancer and hereditary effects increases linearly with radiation dose.^6^ These radiation‐induced effects are known as deterministic and stochastic effects.^7^ High acute doses in excess of one or two gray (Gy) or Sievert (Sv) cause substantial cell death.^7^ ^(p.11–12)^ It is expressed as organ and tissue damage, leaving patients exposed to cellular toxicities.^7^, ^8^ At doses below 10 mGy, the probability of deterministic effects is zero; however the threats of stochastic effects are still present.^1^ Stochastic effects, although not acute compared to deterministic effects, still have the potential to cause cancer and hereditary diseases.^7^ Strong epidemiological evidence indicates a linear relationship between cancer induction and radiation dose for intermediate doses ranging approximately from 0.15 to 1.5 Gy.^8^ There are however limitations to the epidemiological data giving rise to uncertainties in quantifying lifetime attributable radiation risk. The American Association of Physicists in Medicine (AAPM) acknowledges that medical imaging procedures should be appropriate and conducted at the lowest radiation dose.^9^ The AAPM emphasises that discussion about risks related to radiation dose from medical imaging procedures should acknowledge the benefits of the procedure to prevent prescribed procedures with clinical benefits being refused.^9^ This has prompted the AAPM to issue a public position statement in this regard stating that the ‘risks of medical imaging at effective doses below 50 mSv for single procedures or 100 mSv for multiple procedures over short time periods are too low to be detectable and may be non‐existent’ and that predictions of hypothetical cancers are harmful and should be discouraged.^9^ Radiographers can prevent unnecessary radiation exposure through justification of requested radiological examinations by ensuring the clinical benefits offset the radiation detriment.^5^, ^10^ Therefore through the practice of justification, the chances of adverse health effects can be reduced or nullified if the requested examination does not meet the clinical merit.

## Australian Regulatory Bodies

The Australian Radiation Protection and Nuclear Safety Agency (ARPANSA)^11^ is the Australian Regulatory Body responsible for radiation protection as mandated by the Australian Government. ARPANSA oversees radiation protection and nuclear safety and regulates medical applications that use ionising radiation. ARPANSA establishes roles and responsibilities and in particular for the radiation medical practitioner, i.e. the radiologist, and the operator, i.e. the radiographer, as being responsible for justification of procedures involving ionising radiation exposure.^11^ ARPANSA further advocates that the ‘Responsible Person’ must have protocols in place to ensure that “no radiation procedure is carried out unless:

(a) it has been justified, either:
  (i) generically or on an individual basis by the radiation medical practitioner, in accordance with clause 3.2.2 depending on the nature of the procedure and the patient; or
  (ii) generically by an acknowledged professional college or authority;
(b) it has been approved for each individual by:
  (i) the radiation medical practitioner; or
  (ii) the operator in accordance with written guidelines established by:
    (a) the radiation medical practitioner; or
    (b) an acknowledged professional college or authority; 11 ^(pg 5)^

The professional body the Royal Australian and New Zealand College of Radiologists (RANZCR) has as its mission to drive the proper and safe use of radiological and radiation oncological medical services for optimum health outcomes.^12^ The current legislative framework governing the practice of radiographers in Australia is set by the regulatory body the Medical Radiation Practice Board of Australia (MRPBA) who has established the scope of professional capabilities^13^ that must be demonstrated by registered radiographers.

## International Regulatory Bodies

International regulatory bodies recommend the practice of justification for all diagnostic practices.^5^ The United Kingdom and European regulatory authority, the Ionising Radiation (Medical Exposure) (Amendment) Regulations 2011, states that justification is a central task which must be completed prior to a medical exposure being made.^14^ Regulation 6 provides specific guidance on ‘justification of individual medical exposures’ and recognises that the medical practitioner takes overall responsibility for justification of radiological requests, and should produce guidelines that must be followed by operators, allowing operators the freedom to exercise professional judgement.^14^ ^(p.18)^ The 2007 recommendations of the ICRP on radiological protection endorses the use of justification of medical procedures and the optimisation of radiological protection as appropriate mechanisms to avoid unnecessary radiation exposure.^1^ This requires radiographers to assess the clinical indications, apply their knowledge of expected yield from examinations, and demonstrate awareness of how results can influence the diagnosis and management of patients.^5^

The “Bonn Call‐for‐Action”^15^ is a joint position statement by the advisory body the International Atomic Energy Agency (IAEA) and the World Health Organization (WHO) which addresses a specific outcome to prioritise radiation protection in medicine. The “Bonn Call‐for‐Action” has highlighted justification as the ‘Action One’ position statement, namely to “Enhance the implementation of the principle of justification” and to ensure that justification is effective, transparent and accountable as part of the normal activities in a radiological practice.^15^ In addition ‘Action 8’ is aimed at ‘Strengthening radiation safety culture in health care’ and is a call to foster closer co‐operation between different professional associations.^15^ In the context of Australia, this should be a call for RANZCR and the Australian Society of Medical Imaging and Radiation Therapy (ASMIRT) formerly known as Australian Institute of Radiography (AIR) to foster closer collaboration to address the issue of radiation protection so that the professions can act in the best interest of the patient.

Australian researchers have expressed differing opinions over concerns of radiographers’ scope of practice and factors that hinder their willingness and ability to carry out justification of radiographic examinations.^16^, ^17^, ^18^ Yielder and Davis^16^ explored key issues characterising the radiography profession's cultures in the United Kingdom, New Zealand and Australia. The authors argued that because of medical dominance, the resultant monopoly by one profession can create conflict in another profession, thus perpetuating a lack of professional autonomy.^16^ Maintaining the hierarchical position by the medical profession can become a significant barrier to professional role advancement. A conforming work culture could result in tendencies where radiographers are unwilling to challenge traditional models and protocols and would be reluctant to challenge requests that are potentially inappropriate.^17^ Lewis et al^18^ suggested that there are a number of contributing factors such as medical dominance and the presence of the private radiology enterprise that affects radiographer's ethical commitments to patients. These factors could give rise to subordination, inferiority and intimidation. Consequently this could induce apathy amongst radiographers^18^ and negatively affect the practice of justification.

The Australian National Audit Office undertook an independent performance audit titled ‘Diagnostic Imaging Reforms’ to address the increase in diagnostic imaging services and the resultant impact on costs. The reform package proposed a focus on ‘improving appropriate requesting’ in order to address the increase in diagnostic imaging services.^19^ ^(P.54)^ It also recognised that incentives for providers of diagnostic imaging services could lead to preferential requesting. Given these concerns, it is therefore also the radiographers’ responsibility as the operator^11^ to ensure that the principles of justification is adhered to as the radiologist‐referring practitioner relationship could have the potential for unethical practice.^18^

Justification is an advocated and obligatory practice for radiographers, but there are still issues limiting its full implementation into clinical practice in Australia. This review will focus on the role of ‘justification’ in radiographic examinations and the barriers preventing its consistent practice.

## Professional Entities

The comparison between the professional capabilities expected of radiographers as outlined by the MRPBA^13^ and previous studies performed in Australian clinical centres demonstrate obvious inconsistencies.^16^, ^17^, ^18^ To gain registration or remain as a registered radiographer, a practitioner must be able to demonstrate the scope of capabilities as identified in the “Professional Capabilities for Medical Radiation Practice” by the MRPBA.^13^ Although the role of a radiographer is mainly based around diagnostic imaging, they also have a responsibility to act as advocates for patients, which should involve intervening when necessary for the protection of patient when applying safe radiation practice.^13^ This requires justification of requested examinations where medical radiation is involved.

In addition to the professional capabilities framework, the MRPBA released the 2014 ‘Code of Conduct’ directive for medical radiation practitioners (MRPs)^20^ that considers good practice as understanding and applying risk minimisation to practice. It is worth noting that the MRPBA ‘Code of Conduct’ for MRPs expects practitioners to practice the safe use of radiation so that the best possible outcomes for patients are delivered.^20^ ^(p.8)^ Radiographers should justify requested diagnostic examinations and optimise radiation protection for patients while still achieving good diagnostic information using the lowest possible radiation dose.

## Principles of Radiological Protection

ARPANSA advocates that no medical radiation procedure should be carried out unless justified by the radiation medical practitioner and approved by the operator in accordance with agreed protocols before the diagnostic procedure is commenced.^11^ The MRPBA's ‘Code of Conduct in respect to ‘Radiation protection’^20^ ^(p.27)^ expects from practitioners to exhibit good practice by justifying the net benefit of the investigation and in particular with young or pregnant patients. The principle of justification is further endorsed by the MRPBA's expectation of professional capabilities.^13^ Specifically Domain 4 states that ‘Safe radiation practice requires the practitioner to review the referral and procedures to ensure appropriate justification, optimisation and protection’.^13^ ^(P. 7)^ Both the MRPBA's ‘Code of Conduct’^19^ and its ‘Professional Capabilities’^13^ are therefore complimentary to ARPANSA's Code of Practice RPS14^11^ in providing guidance to radiographers with reference to safe radiation practice in order to ensure appropriate justification of diagnostic investigations. The AIR (2013) state that radiographers must “strive to minimise the radiation dose to the patient” by determining the most appropriate examination and seeking further clarification as required.^2^ ^(p.30)^ Radiographers must therefore be able to justify each imaging procedure requested and ensure it is relevant to answering the clinical question.

The current medico‐legal framework in Australia illustrates the fundamental importance that radiographic justification has on radiation safety, risk management and critical thinking. Radiographers are the last barrier between patients and radiation and must therefore act as advocates on behalf of patients and protect them from any unnecessary radiation exposure. Failure to do so demonstrates neglect for legal, ethical and professional responsibilities.

## Clinical History Taking

The performance of justification in radiology clinics is limited to the clinical information provided.^21^ However diagnostic imaging referrals quite often lack adequate information making it difficult for radiographers to practice or adequately perform justification.^22^ A Greek study found that inadequate clinical information and poorly justified requests resulted in radiologists being unable to decide if requested examinations were justified.^10^ It is therefore also important to consider the MRPBA's professional capability Domain 5:4a that has as an expectation that registered practitioners should be able to ‘confirm the procedure according to clinical indications’.^13^ ^(p.10)^ This requires the practitioner to review the patient's clinical history and adapt the requested examination accordingly.^13^ Clinical history taking should be given more serious consideration by radiographers as it offers benefits to justification, error prevention and clinical management of patients.^23^, ^24^, ^25^, ^26^, ^27^

Lam, Egan and Baird^23^ argue that radiographers must place more emphasis on recording accurate patient data in order to justify decisions. History taking can help radiographers decide if the radiological examination is warranted and also justifies the radiographer's decision in determining the projections required to answer the clinical question.^26^ Additionally, radiographers who perform clinical assessments also provide radiologists with further information to help construct their reports, ultimately benefiting patient management.^23^, ^24^, ^25^, ^26^ History taking encourages the performance of justification and image optimisation and has through research evidence suggested improvement in the diagnosis and clinical management of patients.^23^, ^26^ History taking and the fact that it is a professional expectation^13^ requires from radiographers to perform this as a consistent process within their daily practice.

The United Nations Scientific Committee on the Effects of Atomic Radiation (UNSCEAR) reported that worldwide Computerised Tomography (CT) scans accounted for 5% of radiological examinations and contributed to 34% of the collective dose.^28^ ^(p.25)^ ARPANSA advocates that the radiation medical practitioner should assess each referral for its appropriateness and optimise the examination protocol by reducing the dose, changing the scanned area or by suggesting a non‐ionising modality to the referrer.^11^ Although there has been no specific research performed on protocolling, it is used amongst radiologists and radiographers mostly for CT in scheduling the specific exam and contrast requirements. Protocolling is therefore a vital function to ensure that the most appropriate exam is performed for the clinical question and can potentially contribute to minimising the radiation exposure to the patient.

Even though justification is recognised by the radiographer professional bodies, the radiology professional body RANZCR does not support radiographers justifying medical radiation exposures.^29^ This was evident from the professional capabilities consultation document submitted to the MRPBA where RANZCR argued that the final decision rest with the radiologists, who also maintain that they hold general supervision over radiographers undertaking CT examinations.^29^

## Referral Guidelines

Internationally, justification is a compulsory and strongly advocated practice for radiographers in many countries and is enforced by each country's own governing body for radiography. To encourage the practice of justification and ensure that patients are being referred for procedures that are appropriate, guidelines have been established by numerous countries which include the Royal College of Radiologists (RCR) in the UK,^30^ the American College of Radiology (ACR)^31^ and the Medical Exposures Directive (MED) in Europe.^32^ Referral guidelines are becoming a mandatory part of practice in the UK, with the RCR publishing their own pocket guidebook titled: ‘Making the best use of a department of clinical radiology’.^30^ A recent study by Richards et al^33^ determined that by using referral guidelines, 58% of unjustified radiographic examinations could be prevented. The ACR's ‘Appropriateness Criteria’^31^ is an American radiation dose reduction strategy that can be adopted for emergency or acute care settings. The MED on medical exposures^32^ aimed to optimise efficacy at reasonable dose to the patient and to reduce the number of unnecessary exposures. Similarly, the Australasian College for Emergency Medicine^34^ developed guidelines on diagnostic imaging suggesting clear imaging pathways for medical emergencies of which implementation is encouraged within radiology departments. Recently RANZCR in its statement on Radiation Safety and Medical Imaging, recommended to enhance the implementation of the principle of justification through referral based guidelines.^35^

Similar to Australia, justification is a problematic practice internationally with various studies showing countries with a significant percentage of unjustified prescribed medical imaging examinations.^36^, ^37^, ^38^ Malone et al^39^ and Dougeni et al^40^ cite the lack of awareness and education from radiographers, radiologists and doctors as factors impeding justification. Malone et al^39^ highlighted in their report that 20–77% of examinations performed were either inappropriate or unjustified. This was due to a lack of awareness of available referral guidelines.^39^ Education of radiation dose and justification criteria has to be reinforced amongst radiologists and physicians, so that justification can result in its intended purpose of eliminating clinically non‐indicated examinations.^40^

## Advanced Practitioner Roles

Radiographers from the UK have recognised advanced practitioner roles where they are employed to share in the task of reporting radiographic images.^41^ Apart from the responsibility of reporting, radiographers must also justify each diagnostic imaging examination to determine its appropriateness.^42^ American radiographers are also participating in similar roles, such as mid‐level practitioner assistant and radiologist assistant roles.^43^ Although these roles are under the supervision of radiologists, radiographers are provided with extended clinical roles with regards to patient assessment and management.^43^ The role of radiographers performing justification is more accepted by radiologists in the United Kingdom, who recognise that sharing and delegating justification roles to suitably‐trained radiographers is important when radiologists are not readily available.^44^ However in Australia radiographers are still struggling to gain recognition from RANZCR^29^ to perform the professional expectation of justification which form part of their scope of normal practice. The Inter‐professional Practice Advisory Team established by the AIR (2012) stated that advanced practice must enhance the safety and quality of patient services to the benefit of all patients.^45^ This has resulted in the AIR (2014) establishing pathways to recognise advanced practice for the Australian Medical radiation Professions.^46^ The AIR guidelines include as one of its characteristics of an advanced practitioner to be that of a practitioner who provides optimal, expert, and contextual patient care.^46^

## Benefits of Justification

While the initiatives for performing justification are many, undoubtedly the most important benefit of justification is the radiation protection for patients. Limiting the effective dose received by patients is an essential element for medical practice since medical use of radiation accounts for more than 99.9% of radiation exposure worldwide.^3^ Osman and Williams^47^ recommended that the lateral chest should not be performed routinely unless justified, which could result in a mean effective dose reduction of 0.11 mSv.^48^ to the patient. An approach should be adopted that if a procedure is not justified, then it should not be performed.^49^ Justification should result in patients only undergoing examinations relevant to their presenting features and circumstances,^39^ thereby reducing patient dose by eliminating unnecessary examinations and ultimately contributing to improved patient management. When due consideration is given to justification, it could translate to improved access to clinically relevant procedures with associated diagnostic and economic benefits.^39^ Justification of examinations would further enhance effective communication and assist risk management when relevant radiological information is made consistently available to radiologists.^26^

Eliminating unjustified examinations within clinical practice should contribute towards reducing costs to the health care system.^22^, ^26^, ^49^ The implications for the radiology department when justification is not performed are the ineffective use of staff time and waste of resources.^22^ Doyle et al^50^ reported that the anteroposterior view rarely added significant information when taking follow‐up views of anterior cervical plating and that its elimination could reduce expenses if a single projection was excluded and also lead to a reduction in radiation exposure to the patient. Furthermore, Cooney and Campbell^51^ reported potential savings in the UK as none of the patients in their study had their management changed following the post‐operative hip x‐ray exam. Therefore exclusion of the post‐operative hip x‐ray exam should be considered when applying the principles of justification.

Instead of questioning and challenging unethical practice as expected with justification, radiographers have become subservient to the radiologist. Performing justification consistently therefore remains challenging for radiographers. However justification practiced consistently in Australia could present an opportunity for radiographers to raise their professional profile, professional satisfaction and quality of practice by having an increased influence on patient management.^26^ Considering the practice of justification is based on the application of evidence‐based research, justification should encourage critical thinking. This allows for the development of new practices and protocols, benefiting patients in the future.^17^, ^51^

## Evidence‐Based Practice (EBP) and Justification

The MRPBA has a professional expectation^13^ that MRPs will engage in EBP and apply critical thinking and appraisal of literature and evidence. The AIR^2^ advocates that practitioners must critically evaluate published research and consider its application to clinical practice. When justifying radiological examinations, the practitioner needs to consider a range of variables, with the outcome informed by valid evidence.^4^, ^30^, ^34^, ^38^ Evidence‐based rules’ such as the ‘Ottawa Rule for x‐rays of the knee’^52^ can inform clinical decision‐making and justification of examinations. Upton et al^53^ argues that radiographers lack the skills, knowledge and attitude needed for EBP. Sim and Radloff^17^ reports that radiographers are unable to adopt EBP approaches due to a strict adherence to protocols. Incorporating EBP as part of clinical decision‐making will help ease the process of justification by radiographers when supported by research‐based evidence.^52^

The Society and College of Radiographers (SCoR)^54^ has identified barriers to research which falls into two main categories, namely ‘research capacity and capability’ and ‘funding’. Identified strategic drivers to aid research in the radiography profession in the UK include developing a research culture and ensuring that research is put into practice.^54^ Yielder and Davis^16^ suggest that radiographers should demonstrate a culture of openness, participate with other professions and value the importance of research and education. It is encouraging to note that the AIR identifies evidence‐based judgement as a key characteristic of an ‘AIR Advanced Practitioner’.^46^ It would be worthwhile for Australian radiographers to consider the benefits of radiographer‐performed research as it will encourage ‘critical thinkers’ and ‘initiators’^16^ which will result in improved quality of service to patients when they are referred to the radiology department.

## Conclusion

The practice of justification has sufficient clinical merit and with the current medico‐legal framework requires radiographers to perform justification prior to every examination. However this is unfortunately not a consistent practice across Australia. Due to barriers within the radiology department such as medical dominance and workplace culture, the practice of justification amongst radiographers is inhibited. Overcoming these issues can be achieved by radiographer research participation, changes to workplace culture and clinical history taking. The use of referral guidelines in radiology departments must also be encouraged in Australia as it has been proven to help guide decision making and decrease the amount of unjustified radiographic examinations. Radiographers who are the apparent gate‐keepers between the patient and unjustified ionising radiation, should be capable of informing the radiologist or referring physician if referrals are deemed unjustified. Since justification is a fundamental principle of radiation protection, it will help to prevent unnecessary radiation exposure by safeguarding patients from unjustified radiological examinations. Radiographers who actively participate in the decision‐making process of justification of an examination, will ultimately contribute towards improved patient care and management.

## Conflict of interest

The authors declare no conflict of interest.
